# Sinapic Acid Controls Inflammation by Suppressing NLRP3 Inflammasome Activation

**DOI:** 10.3390/cells10092327

**Published:** 2021-09-06

**Authors:** Eun Hye Lee, Jin Hak Shin, Seon Sook Kim, Su Ryeon Seo

**Affiliations:** Department of Molecular Bioscience, College of Biomedical Science, Institute of Bioscience & Biotechnology, Kangwon National University, Chuncheon 24341, Korea; leh8902@kangwon.ac.kr (E.H.L.); wlsgkr1876@kangwon.ac.kr (J.H.S.); painniche@kangwon.ac.kr (S.S.K.)

**Keywords:** NLRP3 inflammasome, sinapic acid, bone marrow-derived macrophages, inflammation, natural compound

## Abstract

A natural phenolic acid compound, sinapic acid (SA), is a cinnamic acid derivative that contains 3,5-dimethoxyl and 4-hydroxyl substitutions in the phenyl ring of cinnamic acid. SA is present in various orally edible natural herbs and cereals and is reported to have antioxidant, antitumor, anti-inflammatory, antibacterial, and neuroprotective activities. Although the anti-inflammatory function of SA has been reported, the effect of SA on the NOD-like receptor pyrin domain-containing 3 (NLRP3) inflammasome has not been explored. In the present study, to elucidate the anti-inflammatory mechanism of SA, we examined whether SA modulates the NLRP3 inflammasome. We found that SA blocked caspase-1 activation and IL-1β secretion by inhibiting NLRP3 inflammasome activation in bone marrow-derived macrophages (BMDMs). Apoptosis-associated speck-like protein containing CARD (ASC) pyroptosome formation was consistently blocked by SA treatment. SA specifically inhibited NLRP3 activation but not the NLRC4 or AIM2 inflammasomes. In addition, SA had no significant effect on the priming phase of the NLRP3 inflammasome, such as pro-IL-1β and NLRP3 inflammasome expression levels. Moreover, we found that SA attenuated IL-1β secretion in LPS-induced systemic inflammation in mice and reduced lethality from endotoxic shock. Our findings suggest that the natural compound SA has potential therapeutic value for the suppression of NLRP3 inflammasome-associated inflammatory diseases.

## 1. Introduction

Sinapic acid (SA, 3,5-dimethoxy-4-hydroxycinnamic acid) is a polyphenol compound commonly present in edible food plants such as wheat, rice, spices, oil seeds, citrus fruits, vegetables, and cereals [1]. SA has been used in traditional Chinese remedies and is considered to act as a potent antioxidant and radical scavenger [2,3]. SA more efficiently inhibited the formation of 3-nitrotyrosine than standard antioxidants, such as ascorbic acid and a-tocopherol [4,5]. SA can scavenge peroxynitrite (ONOO^−^), which is formed from a reaction between superoxide and nitric oxide and can be used for cellular defense against oxidative-induced diseases [6]. SA has been reported to have antiproliferative and apoptotic effects on T47D breast cancer cells [7]. The neuroprotective function of SA and its derivatives has also been reported [8,9,10]. SA shows anxiolytic activity in mice via the GABAergic neurotransmitter system [8]. Sinapine, a derivative of SA, has been reported to have acetylcholine esterase (AChE) inhibitory activity in a reversible binding to AChE [10]. SA has GABA_A_ receptor antagonistic properties and shows a neuroprotective effect on kainic acid (KA)-induced hippocampal brain damage in mice [9]. Lee reported the anti-inflammatory effect of SA in a mouse colitis model, using 2,4,6-trinitrobenzene sulfonic acid (TNBS) [11]. The mechanism by which SA prevents inflammatory colitis involves inhibiting the expression levels of malondialdehyde (MDA), tumor necrosis factor-a (TNF-α), and myeloperoxidase (MPO) [11]. SA shows anti-inflammatory activities in the RAW 264.7 cell line by suppressing lipopolysaccharide (LPS)-induced nitric oxide (NO), prostaglandin E2 (PGE2), TNF-α, and IL-1β production. SA consistently improved serotonin-induced paw edema in mice [12].

Inflammasomes are multimeric protein complexes that initiate inflammation in response to either pathogen-associated molecular patterns (PAMPs) or danger-associated molecular patterns (DAMPs) [13,14]. The NOD-like receptor (NLR) family of inflammasomes includes the nucleotide-binding oligomerization (NOD)-, leucine-rich repeat (LRR)-, and pyrin domain-containing 3 (NLRP3) and NOD-, IRR-, and CARD-containing 4 (NLRC4) inflammasomes [15,16]. Absent in melanoma 2 (AIM2) inflammasomes are non-NLR proteins that sense cytosolic double-stranded DNA [17]. Upon sensing certain stimuli, canonical inflammasomes (NLRP3, NLRC4, and AIM2) undergo self-oligomerization to recruit apoptosis-associated speck-like proteins, containing CARD (ASC) and pro-caspase-1, which leads to the processing of pro-IL-1β and pro-IL-18 into active IL-1β and IL-18, respectively. Recent reports have suggested that the noncanonical inflammasome mediates the innate immune response to the invasion of Gram-negative bacteria [18,19]. Caspase-11 (4/5 in humans) functions as a signal sensor by directly interacting with the intracellular lipopolysaccharide (LPS) to form an active caspase-11 noncanonical inflammasome. Among inflammasomes, the NLRP3 inflammasome has been extensively studied because it is activated by a wide range of signals derived from pathogens as well as hosts and has been linked to the pathogenesis of diverse human diseases [14,20]. The activation of the NLRP3 inflammasome occurs in a two-step mechanism. In the priming step, NLRP3 and pro-IL-1β are transcriptionally upregulated by nuclear factor-κB (NF-κB) signaling in response to LPS-binding to Toll-like receptor 4 (TLR4) [21]. In the second step, the NLRP3 inflammasome is assembled and activated by ATP (P2X7 receptor) or the pore-forming toxin nigericin to generate active caspase-1 for the processing of pro-IL-1β [22]. Pharmacological inhibition or genetic deficiency of the NLRP3 inflammasome shows reduced inflammation in animal models [23,24,25]. Consequently, finding novel natural NLRP3 inflammasome inhibitors is important for the design of safe and effective therapeutics for the treatment of inflammasome-related diseases.

In the present study, we investigated the possibility that SA modulates the NLRP3 inflammasome and provide biochemical evidence that SA regulates inflammatory signaling by suppressing NLRP3 inflammasome activation in vitro and in vivo.

## 2. Materials and Methods

### 2.1. Materials

Sinapic acid (SA, 3,5-dimethoxy-4-hydroxycinnamic acid), LPS, and ATP were purchased from Sigma-Aldrich (St. Louis, MO, USA). Nigericin was purchased from Tocris (Bristol, UK). Poly (dA:dT) and *Salmonella* *typhimurium* flagellin were purchased from InvivoGen (San Diego, CA, USA). Anti-IL-1β antibody (AF-401-NA) was purchased from R&D Systems (Minneapolis, MN, USA). The anti-caspase-1 (AG-20B-0042), anti-NLRP3 (AG-20B-0014), and anti-ASC (AG-25B-0006) antibodies were purchased from AdipoGen Life Science (San Diego, CA, USA). Anti-IL-6 (12912), anti-phospho-STAT3 (9145), anti-phospho-IκBα (9246), anti-phospho-JNK (4668), and anti-phospho-ERK (9106) antibodies were purchased from Cell Signaling Technology (Danvers, MA, USA). The anti-β-actin antibody (sc-47778) was purchased from Santa Cruz Biotechnology (Dallas, TX, USA). All culture reagents were purchased from Thermo Fisher Scientific (Waltham, MA, USA).

### 2.2. Mice

Eight-week-old male C57BL/6 mice were obtained from Orient Bio Inc. (Seongnam, Korea), and the mice were maintained at the Animal Center of Kangwon National University in a controlled environment. All experiments were approved by the Institutional Animal Care and Use Committee (IACUC, KW-201026-1, Kangwon National University, Chuncheon, Korea).

### 2.3. Cell Culture

Bone marrow-derived macrophages (BMDMs) were prepared as described previously [26]. Progenitor cells were isolated from eight-week-old C57BL/6 mice and differentiated into BMDMs in 30% L929 cell-conditioned medium (LCCM) for 7 days [27,28]. For the preparation of LCCM, 4.7 × 10^5^ L929 cells were plated and cultured in a 75-cm^2^ flask containing 50 mL DMEM for 7 days. The culture supernatant was collected and filtered through a 0.45-μm filter. LCCM was kept at −20 °C until use. The differentiated BMDMs were cultured in DMEM containing 10% fetal bovine serum, 30% LCCM, 100 U/mL penicillin, and 100 μg/mL streptomycin. RAW264.7 macrophages were obtained from the American Type Culture Collection (Rockville, MD, USA) and cultured in DMEM, containing 10% FBS, 100 U/mL penicillin, and 100 μg/mL streptomycin. All cells were maintained at 37 °C in a humidified 5% CO_2_ incubator.

### 2.4. MTT Assay

BMDMs were treated with SA at the indicated times or concentrations. The cells were incubated with MTT (Sigma-Aldrich, St. Louis, MO, USA) at a final concentration of 1 mg/mL at 37 °C. After 1 h, MTT formazan was dissolved in DMSO, and the absorbance was measured at 570 nm. The viability of the cells was calculated as the percentage relative to the control cells.

### 2.5. Western Blot Analysis

BMDMs were primed with LPS (500 ng/mL) for 3 h and then treated with or without SA (100 μM and 200 μM) for 30 min. For the analysis of NLRP3 inflammasome activation, cells were subsequently incubated with either ATP (5 mM) or nigericin (10 μM) for 1 h. For the NLRC4 inflammasome activation, flagellin (1 μg/mL) was added to cells for 4 h. To activate AIM2, the poly (dA:dT) (1 μg/mL) was transfected with Lipofectamine 3000 for 6 h. The culture supernatant (Sup, 400 μL) was collected from the 12-well plates and centrifuged at 500× *g* for 5 min to remove detached cells. The cells were resuspended in lysis buffer containing 20 mM Tris-Cl (pH 7.9), 150 mM NaCl, 1% Nonidet P-40, 10% glycerol, 1 mM EGTA, 10 mM NaF, protease inhibitor, 1 mM Na_3_VO_4_, and 0.2 mM phenylmethylsulfonyl fluoride (PMSF) and incubated on ice for 30 min. After centrifugation, the lysates were collected, and the proteins were separated via SDS-PAGE. The proteins were transferred onto nitrocellulose membranes, and the membranes were blocked with 5% skim milk in TBS-T buffer. The membranes were incubated with the indicated primary antibodies (1:1000) overnight, and then washed with TBS-T buffer. The membranes were incubated with secondary antibodies (1:5000) for 1 h. The bands were visualized with enhanced chemiluminescence solution.

### 2.6. ELISA

Blood samples were centrifuged at 800× *g* for 20 min. The supernatant (plasma) was transferred into a new tube and the concentrations of IL-1β and IL-6 were measured in accordance with the manufacturer’s instructions (R&D Systems, Minneapolis, MN, USA).

### 2.7. Real-Time PCR

Real-time PCR was performed as described previously [26]. cDNA was synthesized with M-MLV reverse transcriptase (Promega, Madison, WI, USA) according to the manufacturer’s protocol and then amplified with SYBR Green Master Mix (TOYOBO, Osaka, Japan), using the following primers: *IL-1β*, 5′-ACCTGTTCTTTGAGGCTGAC-3′ (forward) and 5′-CTTCTTTGGGTATTGTTTGG-3′ (reverse); *IL-6*, 5′-AGTTGCCTTCTTGGGACTGA-3′ (forward) and 5′-TTCTGCAAGTGCATCATCGT-3′ (reverse); *NRLP3*, 5′-ACCTGTTCTTTGAGGCTGAC-3′ (forward) and 5′-CTTCTTTGGGTATTGTTTGG-3′ (reverse); *TNF-α*, 5′-TAGCCCACGTCGTAGCAAAC-3′ (forward) and 5′-GGAGGCTGACTTTCTCCTGG-3′ (reverse); and *β-actin*, 5′-AGAGGGAAATCGTGCGTGAC-3′ (forward) and 5′-CGATAGTGATGACCTGACCGT-3′ (reverse). The values were analyzed using CFX Manager™ (Bio-Rad, Hercules, CA, USA). All samples were run in triplicate and changes in target mRNA expression were normalized to β-actin.

### 2.8. Reporter Gene Assay Analysis

RAW264.7 cells were cotransfected with NF-κB-luc reporter vector and control *Renilla* luciferase reporter vector using the Lipofectamine 3000 method (Thermo Fisher Scientific, Waltham, MA, USA). Luciferase activity was measured using a dual luciferase assay system (Promega, Madison, WI, USA).

### 2.9. Immunofluorescence

BMDMs were cultured on poly-l-lysine-coated coverslips. The cells were primed with LPS (500 ng/mL) for 3 h. The cells were pretreated with SA (200 μM) for 30 min and then incubated with either ATP (5 mM) or nigericin (10 μM) for 1 h. The cells were then fixed with 3.7% formaldehyde in PBS for 15 min and permeabilized with 0.2% Triton X-100 in PBS for 10 min. The samples were blocked with 3% bovine serum albumin (BSA) for 30 min and incubated with anti-ASC antibody overnight at 4 °C, followed by incubation with Alexa-Fluor-555- conjugated secondary antibody (A21429) (Thermo Fisher Scientific, Waltham, MA, USA) for 1 h. After washing, the cells were incubated with DAPI (Sigma-Aldrich, St. Louis, MO, USA) in PBS for 5 min. The coverslips were mounted and analyzed using fluorescence microscopy.

### 2.10. In Vivo LPS Injection

Mice were pretreated with SA (20 mg/kg) intraperitoneally (i.p.) for 3 h before the LPS injection. Control mice received an equal volume of DMSO. After 3 h, LPS (10 mg/kg) was injected i.p. for 6 h before blood sampling. Control mice received an equal volume of normal saline. Blood was obtained by cardiac puncture and collected in anticoagulant-treated tubes. The serum samples were stored at −20 °C until use. Liver, lung, and spleen tissues were homogenized in ice-cold lysis buffer and centrifuged to collect the supernatant. The supernatants were analyzed by Western blot analysis. To measure endotoxic lethality, the nine-week-old male mice (5–8/group) were pretreated with SA (20 mg/kg) for 3 h before LPS (10 mg/kg) injection and were monitored every 12 h for 3 days.

### 2.11. Statistics

Densitometric scans of the Western blot analyses were quantified using ImageJ software (version 1.52a; NIH, Bethesda, MD, USA). All data analyses were performed using GraphPad Prism software (version 5.01; GraphPad Software, Inc., San Diego, CA, USA) and are shown as the means ± SD. Differences between the experimental group and the control group were analyzed using Student’s *t*-test. Comparisons between multiple groups were analyzed using ANOVA, followed by Bonferroni post hoc tests. A value of *p* < 0.05 was considered statistically significant. * *p* < 0.05; ** *p* < 0.01; *** *p* < 0.001.

## 3. Results

### 3.1. Dose Optimization of SA in Bone Marrow-Derived Macrophages (BMDMs)

**Figure 1:**
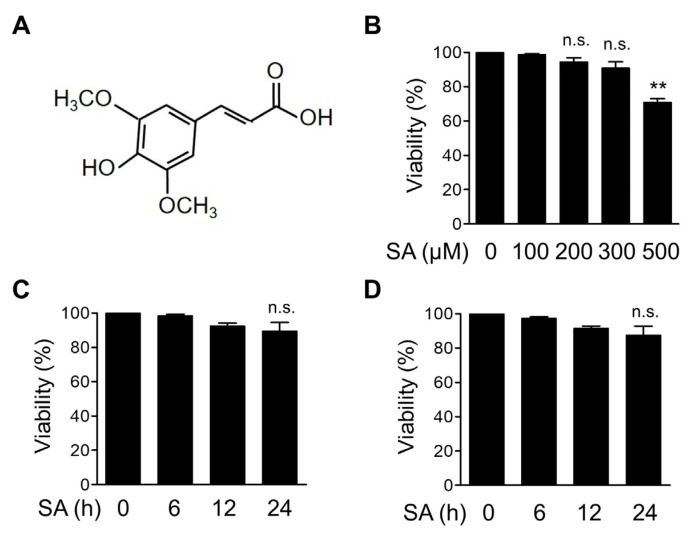
In vitro cytotoxicity analysis of SA in BMDMs. (**A**) Chemical structure of sinapic acid (SA). (**B**) Mouse BMDMs were treated with the indicated concentrations of SA for 12 h, and cell viability was measured using the MTT assay. (**C**,**D**) Mouse BMDMs were treated with 100 μM SA (**C**) and 200 μM SA (**D**) for the indicated times. Cell viability was measured using the MTT assay. The graphs are presented as the means ± SD of three independent experiments. ** *p* < 0.01; n.s., nonsignificant.

To observe the effect of SA on inflammatory signaling pathways, we first determined the noncytotoxic concentration of SA in mouse BMDMs. The structure of SA is depicted in Figure 1A. BMDMs were treated with increasing concentrations of SA for 12 h, and cell viability was measured using the MTT assay (Figure 1B). We found that cell viability was not significantly altered by SA at concentrations up to 300 μM for 12 h (Figure 1B). We then selected 100 and 200 μM SA and treated BMDMs for up to 24 h. We found that cytotoxicity was not significantly affected at these concentrations for up to 24 h (Figure 1C,D). Based on these results, we used 100 and 200 μM SA for all subsequent experiments in BMDMs.

### 3.2. SA Inhibits NLRP3 Inflammasome Activation

**Figure 2:**
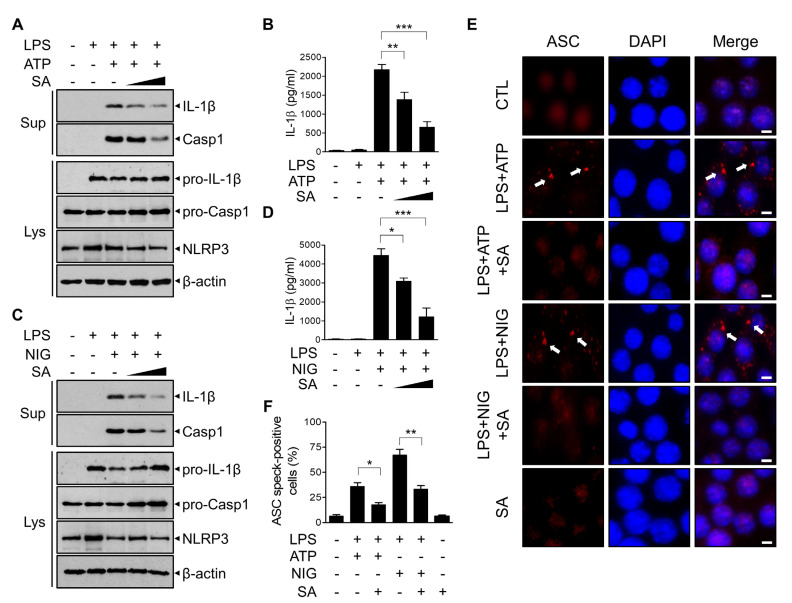
Inhibitory effects of SA on NLRP3 inflammasome activation. (**A**,**C**) Mouse BMDMs were primed with LPS (500 ng/mL) for 3 h and then treated with SA (100 μM and 200 μM) for 30 min before either ATP (5 mM) or nigericin (10 μM) incubation for 1 h. Culture supernatants (Sup) and cell extracts (Lys) were immunoblotted with the indicated antibodies. (**B**,**D**) The secreted IL-1β level in the supernatant was measured via ELISA. (**E**) Representative immunofluorescence images using an anti-ASC antibody (red). DAPI was used to show nuclei (blue). The images were captured separately and merged. Scale bar = 20 mM. (**F**) Percentages of cells with an ASC speck relative to the total number of cells from 5 random fields. The graphs are presented as the means ± SD of three independent experiments. * *p* < 0.05; ** *p* < 0.01; *** *p* < 0.001.

To evaluate the potential effect of SA on NLRP3 inflammasome activation, we analyzed the expression of components of the NLRP3 inflammasome in BMDMs. BMDMs were primed with LPS and subsequently triggered by ATP, which has been reported to activate the NLRP3 inflammasome through the opening of the P2X7 receptor. As shown in Figure 2A, SA treatment inhibited the secretion of the ATP-driven mature form of IL-1β in a concentration-dependent manner. The secreted active caspase-1 level was consistently inhibited by SA treatment (Figure 2A). The decreased secretion of IL-1β protein was further confirmed using ELISA (Figure 2B). To confirm the effect of SA on NLRP3 inflammasome activation, BMDMs were triggered using another canonical NLRP3 inflammasome activator, nigericin, which functions as a lipophilic ionophore. As shown in Figure 2C, SA consistently inhibited nigericin-induced bioactive IL-1β and caspase-1 secretion in a concentration-dependent manner. ELISA results confirmed the decreased IL-1β protein level by SA treatment (Figure 2D). We next investigated whether SA inhibits ASC pyroptosome formation. To monitor ASC pyroptosomes, we visualized ASC specks by means of immunofluorescence staining. As shown in Figure 2E,F, the number of ASC speck-containing cells was increased by ATP or nigericin stimulation and was significantly reduced by SA pretreatment (Figure 2E,F). Taken together, these results indicate that SA exerts an inhibitory effect on NLRP3 inflammasome activation.

### 3.3. SA Has No Effect on the Transcription of Proinflammatory Mediators

**Figure 3:**
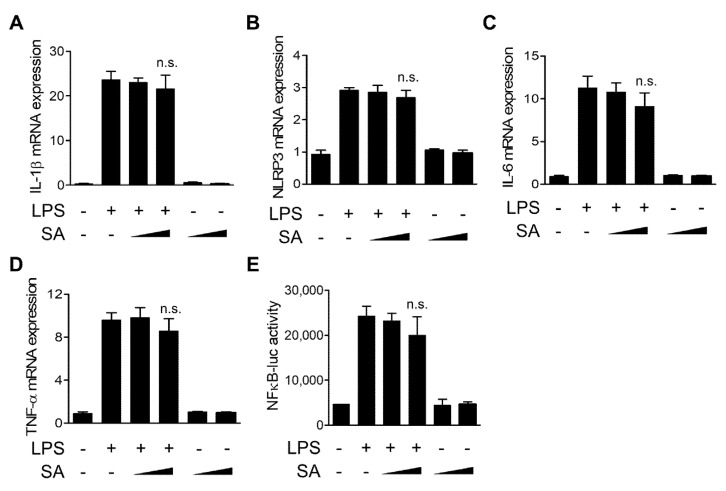
Effects of SA on the transcription of proinflammatory mediators. (**A**–**D**) Mouse BMDMs were pretreated with SA (100 μM and 200 μM) for 30 min and then incubated with LPS (500 ng/mL) for 6 h. IL-1β, NLRP3, IL-6, and TNF-α mRNA levels were measured using qRT-PCR. (**E**) RAW264.7 cells were cotransfected with the NF-κB-luciferase reporter vector (NF-κB-luc) and *Renilla* luciferase reporter vector. After 24 h, the cells were pretreated with SA (100 μM and 200 μM) for 30 min and then incubated with LPS (500 ng/mL) for 6 h. The luciferase activity was measured and normalized to *Renilla* reporter activity. The graphs are presented as the means ± SD of three independent experiments. n.s., nonsignificant.

We next evaluated the effects of SA on the transcription of inflammatory mediators using quantitative real-time PCR. As shown in Figure 3A,B, LPS-induced increases in IL-1β and NLRP3 mRNA levels, components of the NLRP3 inflammasome, were not changed by SA treatment. Furthermore, SA treatment did not cause a decrease in mRNA levels of inflammasome-independent cytokines, such as IL-6 and TNF-α (Figure 3C,D). We next used reporter analysis to measure the effect of SA on the activation of the nuclear factor-κB (NF-κB) transcription factor, which is involved in the transcription of many inflammatory cytokine genes in response to LPS. As shown in Figure 3E, NF-κB-dependent gene transcription in response to LPS was not suppressed by SA treatment. Collectively, these results indicate that SA has no significant effect on the transcription of LPS-induced inflammatory mediators.

### 3.4. SA Has No Effect on the Priming Phase of the NLRP3 Inflammasome

**Figure 4:**
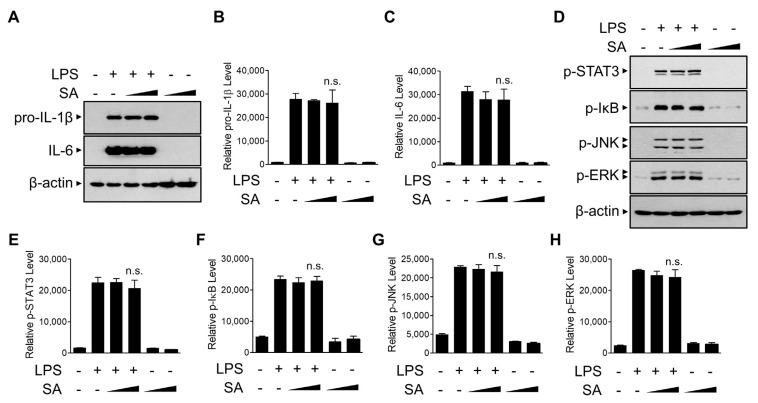
Effects of SA on priming of the NLRP3 inflammasome. (**A**) Mouse BMDMs were pretreated with SA (100 μM and 200 μM) for 30 min and then incubated with LPS (500 ng/mL) for 6 h. The cell lysates were immunoblotted with the indicated antibodies and quantified (**B**,**C**). (**D**) Mouse BMDMs were pretreated with SA (100 μM and 200 μM) for 30 min and then incubated with LPS (500 ng/mL) for 30 min. The cell lysates were immunoblotted with the indicated antibodies and quantified (**E**–**H**). The graphs are presented as the means ± SD of three independent experiments. n.s., nonsignificant.

We next examined the effect of SA on the priming phase of NLRP3 inflammasome activation. We monitored the effect of SA on pro-IL-1β protein levels, the precursor of IL-1β. As shown in Figure 4A,B, SA had no significant effect on LPS-induced pro-IL-1β protein levels, indicating that the inhibitory effect of SA on the secretion of the active IL-1β protein was not caused by the decrease in pro-IL-1β protein levels. Furthermore, SA did not alter inflammasome-independent IL-6 protein levels (Figure 4A,C). We next examined the effect of SA on the activation of diverse known intracellular signaling regulators involved in the priming phase of the NLRP3 inflammasome [29]. Because LPS is known to activate signal transducer and activator of transcription 3 (STAT3), mitogen-activated protein kinases (MAPKs), and NF-κB signaling to induce various cytokine gene expressions, we measured the activation of STAT3, IκB, JNK, and ERK using phosphorylation-specific antibodies (Figure 4D–H). SA did not suppress any of these LPS-induced phosphorylation of inflammatory regulators, indicating that SA does not modulate the LPS-induced priming phase of the NLRP3 inflammasome in BMDMs.

### 3.5. SA Has No effect on AIM2 or NLRC4 Inflammasomes

**Figure 5:**
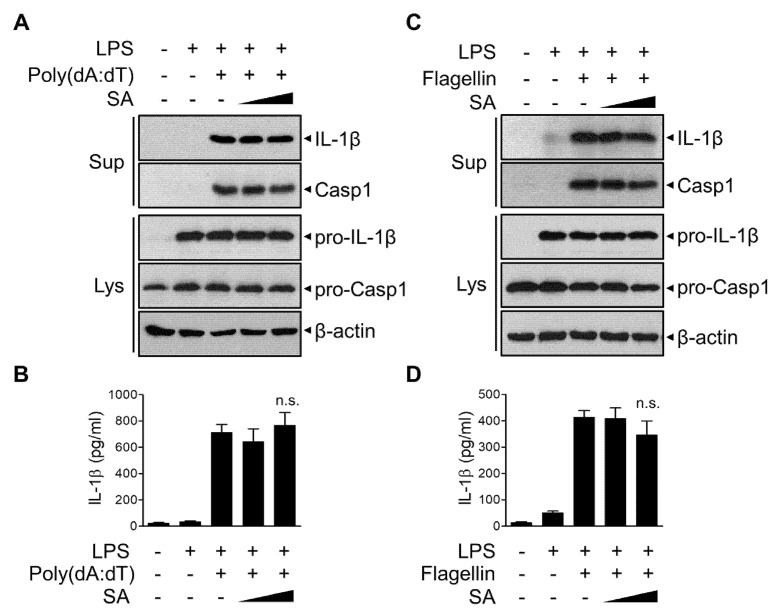
Effects of SA on AIM2 and NLRC4 inflammasomes. (**A**–**D**) Mouse BMDMs were primed with LPS (500 ng/mL) for 3 h and then treated with SA (100 μM and 200 μM) for 30 min before poly (dA:dT) (1 μg/mL) transfection with Lipofectamine 3000 for 6 h (**A**,**B**) or flagellin treatment (1 μg/mL) for 4 h (**C**,**D**). (**A**,**C**) Culture supernatants (Sup) and cell extracts (Lys) were immunoblotted with the indicated antibodies. The cell lysates were immunoblotted with the indicated antibodies. (**B**,**D**) The secreted IL-1β level in the supernatant was measured via ELISA. The graphs are presented as the means ± SD of three independent experiments. n.s., nonsignificant.

To investigate the possible effect of SA on other inflammasomes, LPS-primed BMDMs were stimulated with poly (dA:dT) to activate the AIM2 inflammasome (Figure 5A,B). The release of poly (dA:dT)-induced active IL-1β and caspase-1 was not significantly suppressed by SA treatment (Figure 5A,B). Furthermore, SA had no effect on the release of IL-1β or caspase-1 following flagellin treatment to activate the NLRC4 inflammasome (Figure 5C,D). These results indicate that SA specifically inhibits NLRP3 inflammasome and prevents subsequent IL-1β secretion.

### 3.6. SA Reduces LPS-Induced Systemic Inflammation In Vivo

**Figure 6:**
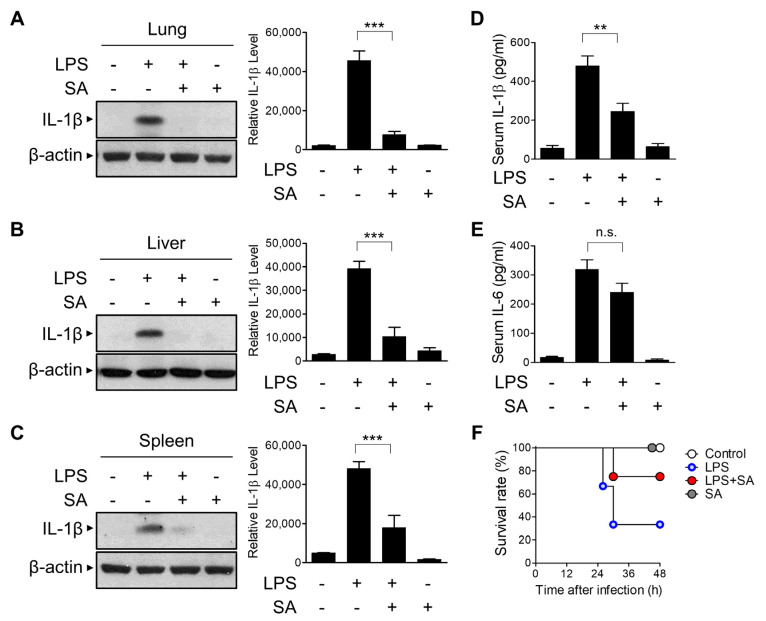
Inhibitory effects of SA on LPS-induced systemic inflammation. C57BL/6 mice were preinjected with SA (10 mg/kg) for 3 h before LPS (10 mg/kg) i.p. injection. The mice were sacrificed 6 h after LPS injection, and whole blood, liver, lung, and spleen were collected. The tissue samples were homogenized and immunoblotted with the indicated antibodies (**A**–**C**). IL-1β (**D**) and IL-6 (**E**) protein levels in serum were detected via ELISA. (**F**) The survival rate was recorded at the indicated times. Comparison of survival curves was performed using the log rank test. The graphs are presented as the means ± SD of 5–8 mice/group. ** *p* < 0.01; *** *p* < 0.001; n.s., nonsignificant.

We next evaluated the effect of SA on NLRP3 inflammasome activation in vivo using a mouse model. IL-1β secretion in LPS-induced systemic inflammation has been shown to be NLRP3 inflammasome-dependent [30,31,32]. C57BL/6J mice were injected intraperitoneally (i.p.) with SA before LPS injection, and active IL-1β expression in lung tissues was evaluated using Western blot analysis (Figure 6A). SA pretreatment blocked the secretion of active IL-1β in lung tissues (Figure 6A). The suppressive effect of SA on LPS-induced IL-1β expression was similarly observed in liver and spleen tissues (Figure 6B,C). To further characterize the effect of SA on LPS-induced systemic inflammation, serum IL-1β levels were monitored via ELISA. As shown in Figure 6D, SA significantly reduced LPS-induced IL-1β serum levels. However, SA had no effect on IL-6 levels compared with the LPS-treated group (Figure 6E). We next monitored the effect of SA on LPS-induced endotoxic lethality. As shown in Figure 6F, the survival rate was consistently increased in the SA-treated groups compared with the LPS-treated group. Collectively, these results suggest that SA can inhibit inflammatory signaling by suppressing NLRP3-mediated IL-1β secretion in vivo.

## 4. Discussion

In the present study, we identified the natural compound SA as a novel inhibitor of NLRP3 inflammasome activation. SA efficiently inhibited NLRP3 inflammasome activation in vitro and in vivo. The inhibitory effect of SA was specific to NLRP3 but not to NLRC4 or the AIM2 inflammasome. Moreover, SA attenuated endotoxic shock from LPS-induced NLRP3 inflammasome-dependent systemic inflammation in mice by reducing active IL-1β expression levels.

IL-1β is an important cytokine that promotes acute and chronic inflammation in various diseases [33]. The IL-1β precursor is inactive until it is cleaved by caspase-1, a cysteine protease. For the activation of caspase-1, the NLRP3 inflammasome is required. The NLRP3 inflammasome is regarded as an important initiator in diverse human diseases, including type 2 diabetes (T2D), gout, obesity, atherosclerosis, neurodegenerative diseases, cancer, and inflammatory diseases, and has been suggested as a potential target for the treatment of these diseases [34,35,36,37,38]. The soluble decoy IL-1β receptor rilonacept and the neutralizing IL-1β antibody canakinumab have been used for the clinical treatment of cryopyrin-associated autoinflammatory syndrome (CAPS), which is caused by the NLRP3 mutation [33]. Although these have efficacy in the treatment of inflammation, the NLRP3 inflammasome is not the only inflammasome to generate IL-1β. IL-1β can be generated by other inflammasomes or in an inflammasome-independent manner.

In previous reports, a few small molecules, such as MCC950, OLT1177, oridonin, tranilast, and CY-09, were suggested as NLRP3 inflammasome inhibitors via different mechanisms [23,39,40,41]. Small-molecule compounds are generally more cost effective than biological agents [42]. A recent study showed that ginsenoside Rg3, one of the main constituents of *Panax ginseng*, blocks IL-1β secretion and caspase-1 activation through the inhibition of NLRP3 priming and inflammasome activation in human and mouse macrophages [32]. In accordance with these reports, our study suggests that SA acts as an inhibitor of NLRP3 inflammasome activation and prevents IL-1β secretion. It has been reported that SA has an anti-inflammatory effect, but the underlying mechanism has not been well studied. Our results show that SA exerts an anti-inflammatory effect by suppressing NLRP3 inflammasome activation. SA specifically inhibited NLRP3 inflammasome activation, but not priming. SA is one of the polyphenols that is abundantly present in the plant kingdom and can be obtained from diverse sources, such as cereals, fruit, and vegetables [43]. Polyphenols, the structure of which is composed of one or more benzene ring joined to hydroxyl groups, are the most abundant and safe antioxidant phytochemical compounds in the human diet. Several polyphenol compounds have been reported to have anti-inflammatory capacity and effects on the NLRP3 inflammasome in chronic and metabolic diseases. For example, red raspberry crude powder, enriched in polyphenols, suppresses NLRP3 activation and attenuates metabolic abnormalities in diet-induced obese mice [44]. Apigenin partially inhibits NLRP3 and AIM2 inflammasome activation by interrupting the Syk/Pyk2 pathway, but not NLRC4 [45]. Curcumin suppresses NLRP3 expression through the inhibition of TLR4/MyD88/NF-κB signaling [46]. Rosmaric acid suppresses oxidized low-density lipoprotein (oxLDL)-induced NLRP3 inflammasome assembly through the downregulation of thioredoxin-interacting protein (TXNIP), a negative regulator of thiol-reducing thioredoxin (TRX) [47].

Finding new molecules with higher potency but lower toxicity to modulate the NLRP3 inflammasome is an important therapeutic goal for treating inflammation-related diseases. Phytochemically identified compounds such as SA might be good candidates for the design of effective and safe therapeutic applications. Although studies on the toxicity of SA are limited, some studies have analyzed its toxicity in different types of cells. The cytotoxic profiles in V79 Chinese hamster lung fibroblasts and HeLa cells showed no effects on the viability of cells treated with up to 2 mM SA [48]. SA induced cytotoxic effects at a very high concentration above 5 mM in these cells [48]. SH-SY5Y human neuroblastoma cells treated with SA did not show cytotoxic effects up to 100 mg/mL for 24 h [49]. In our results, SA did not induce significant cytotoxicity up to 200 mM for 24 h in mouse BMDMs (Figure 1). However, further in vitro and in vivo investigations will be needed to evaluate the efficacy and toxicity of SA before clinical application.

In conclusion, our study is the first demonstration that SA suppresses NLRP3 inflammasome activation and provides important scientific evidence for the potential application of SA as a therapeutic agent in the treatment of IL-1β-mediated inflammatory diseases.

## Data Availability

Not applicable.
